# Autochthonous Chagas Disease — Missouri, 2018

**DOI:** 10.15585/mmwr.mm6907a4

**Published:** 2020-02-21

**Authors:** George Turabelidze, Archana Vasudevan, Christian Rojas-Moreno, Susan P. Montgomery, Molly Baker, Drew Pratt, Susanne Enyeart

**Affiliations:** ^1^Division of Community and Public Health, Missouri Department of Health and Senior Services, Jefferson City, Missouri; ^2^Division of Infectious Diseases, Department of Medicine, University of Missouri School of Medicine, Columbia, Missouri; ^3^Division of Parasitic Diseases and Malaria, Center for Global Health, CDC.

On December 13, 2017, the Missouri Department of Health and Senior Services (MDHSS) was notified of a suspected case of Chagas disease in a Missouri woman. The patient had donated blood, and laboratory screening revealed antibodies to *Trypanosoma cruzi*, the parasite that causes Chagas disease. Evaluation by physicians found no clinical symptoms consistent with Chagas disease. The patient had no travel history that would have suggested a significant risk for Chagas disease risk and had no occupational exposure to the disease agent. She had never received a blood transfusion or organ transplant. Confirmatory testing of the patient’s serum at CDC for *T. cruzi* antibody was consistent with infection. These findings raise the possibility that the exposure to *T. cruzi* occurred locally (autochthonously) in Missouri. Although the insect vector for the parasite *T. cruzi*, triatomines (commonly known as “kissing bugs”), has been identified previously in Missouri, no locally acquired human cases of Chagas disease have been identified in the state. Health care providers and public health professionals should be aware of the possibility of locally acquired Chagas disease in the southern United States.

## Case Report

In October 2017, a woman aged 53 years visited her local blood donation center to donate blood. On October 25, 2017, she was notified that a screening test (Abbott Prism Chagas; Abbott Laboratories) of the collected blood was positive for antibodies to *T. cruzi*. The follow-up confirmatory multistep enzyme strip immunoassay test (Abbott ESA Chagas; Abbott Laboratories) performed on November 8, 2017, also yielded a positive result. The patient was referred by her physician to an infectious disease specialist for further evaluation.

The patient reported no known triatomine bites. Her travel history was remarkable for a trip to California approximately 28 years earlier, when she crossed the Mexican border for a few hours to go shopping. She also traveled to Florida and Alabama for vacation but could not recall the specific year. She reported no insect bites or any medical complaints during those trips.

The patient underwent diagnostic testing at a commercial laboratory on November 28, 2017, with an enzyme-linked immunosorbent assay (ELISA) for *T. cruzi* immunoglobulin G (IgG), and the results were positive. After discussion with subject matter experts at CDC, confirmatory diagnostic testing was done on September 6, 2018. All diagnostic tests were developed at CDC’s Parasitic Diseases laboratory. A Wiener recombinant antigen enzyme immunoassay (EIA) for *T. cruzi* antibody was positive, and the trypomastigote excreted secreted antigen (TESA) immunoblot assay, a laboratory-developed test, was negative. Because the first two test results were discordant, related to limitations of specificity and sensitivity associated with differences in *T. cruzi* strains endemic in different geographic areas, a third test, a laboratory-developed immunofluorescence assay (IFA) for *T. cruzi* IgG antibody, was conducted, and the result was positive.

Given the potential for *T. cruzi* infection to cause cardiomyopathy, an electrocardiogram was obtained, which showed arrhythmias, including primary atrioventricular block with prolonged PR interval (increased time between the beginning of the P wave and the start of the QRS complex). The patient also underwent echocardiography, which showed mild concentric left ventricular hypertrophy. Both findings were consistent with the chronic phase of infection. After all confirmatory testing, the patient completed a 60-day course of benznidazole (5 mg/kg/day) as treatment for Chagas disease. The patient's blood cell count and liver enzyme levels were monitored closely during her trypanocidal course for treatment side effects.

## Public Health Investigation

The patient had lived in Missouri her entire life and moved to her current county of residence in 1999. From 1999 to 2012, she had lived in three different houses of varying structural integrity and had moved into her current (fourth) house in 2012. The patient reported seeing boxelder bugs, which do not transmit disease, but had never seen a “kissing bug” at any of the four properties. The patient's husband recalled seeing some insects consistent with the digital images of triatomine bug in one of their residences but could not recall specific time and location.

An environmental evaluation was conducted by MDHSS and local public health agency staff members at all four residencies. Brush, woodpiles, and construction debris were found on most properties. Time-worn, nonresidential structures (e.g., sheds) that provided opportunities for animal nesting were also present at all properties. Two of the older residential structures had sufficient external damage to allow easy insect access to the interior. There were crawl spaces under the two houses. No triatomine insects were detected at any of the properties.

## Discussion

The protozoan parasite *T. cruzi* can be transmitted by infected insect vectors, from mother to baby (congenital) and, much less commonly, through organ transplantation or blood transfusion from an infected donor. Transmission through the oral route has also been described (*1*). Mammals, especially rodents and marsupials, are reservoirs of *T. cruzi* in a sylvatic cycle, but humans, dogs, and cats can also serve as reservoirs in areas where the parasite is endemic. Acute *T. cruzi* infection is rarely identified because it usually causes a mild nonspecific illness or is asymptomatic. Without treatment, infection persists for the lifetime of the infected person and can result in gastrointestinal disease or serious cardiac manifestations, including heart failure, stroke, or life-threatening ventricular arrhythmias in approximately 30% of those who are chronically infected (*2*).

An enzootic *T. cruzi* transmission involving at least 11 triatomine species and 24 species of wild animals has been well documented in the southern United States going back approximately 150 years (*3*). Historical records of triatomine findings show species distributed across at least 29 states in the United States (*4*). There are an estimated 300,000 persons with Chagas disease in the United States, but only 28 autochthonous infections had been documented from 1955 to 2015 (*5*). Triatomine species in the United States are primarily sylvatic and typically not found colonizing human dwellings. Prevalence of *T. cruzi* infection in triatomines can vary and transmission is not efficient; the parasite is passed in the triatomine’s feces and infection occurs when feces contaminate a break in the skin or conjunctiva. In Missouri, *Triatoma sanguisuga* vectors have been identified during 2012–2016 and as recently as July 2019 (MDHSS surveillance, unpublished data, 2019) (*6*). In 2018, a single finding of *T. lecticularia* in Missouri was confirmed by molecular typing at CDC.

Blood donor screening for *T. cruzi* antibodies in the U.S. blood supply was first implemented in 2007 (*3*). Positive results from blood donor screening for *T. cruzi* antibodies should be followed by diagnostic testing. Diagnosis of chronic Chagas disease is based on positive results from at least two serologic tests that use different techniques and different antigen preparations because no single test is sufficiently sensitive and specific for diagnosis.* Commonly used techniques include ELISA using recombinant antigens, TESA, and IFA. This patient’s results were positive with EIA and negative with TESA. CDC’s testing algorithm employs a third test when results of the first two tests are discordant. In the Missouri patient, the IFA result was positive for *T. cruzi* antibody. Based on the patient’s testing results, history and presentation, this patient likely represents the first documented autochthonous case of Chagas disease in Missouri.

Most persons with Chagas disease acquired their infection in the parts of Latin America where Chagas disease is endemic (*5*). Currently, more than a century after its discovery in Latin America, Chagas disease has a global distribution including the United States because of migration from areas with endemic disease. Few cases of locally acquired vectorborne infection have been reported in the United States. The likely autochthonous case described in this report underscores importance of health care provider awareness of possible Chagas disease even in the states considered low risk for this infection and need for the careful consideration of the patient’s history to identify possible risks for *T. cruzi* infection.

**Summary**

**What is already known about this topic?**

Locally acquired cases of Chagas disease are exceedingly rare in the United States. Only 28 autochthonous infections were documented from 1955 to 2015.

**What is added by this report?**

In 2017, a blood donation in Missouri screened positive for antibodies to *Trypanosoma cruzi*, the parasite that causes Chagas disease. Based on the epidemiologic, clinical, and laboratory data, the reported case likely represents the first documented autochthonous case of Chagas disease in Missouri.

**What are the implication for public health practice?**

Although most documented cases are among persons originally from Latin America, health care providers and public health professionals should be aware of the possibility of locally acquired Chagas disease in the southern United States.

## Footnotes

* https://www.cdc.gov/parasites/chagas/health_professionals/dx.html.
